# Nodal root diameter and node number in maize (*Zea mays* L.) interact to influence plant growth under nitrogen stress

**DOI:** 10.1002/pld3.310

**Published:** 2021-03-16

**Authors:** Hannah M. Schneider, Jennifer T. Yang, Kathleen M. Brown, Jonathan P. Lynch

**Affiliations:** ^1^ Department of Plant Science The Pennsylvania State University University Park PA USA; ^2^ Present address: Wellesley College Wellesley MA USA

**Keywords:** maize, nitrogen stress, root cross‐sectional area, root number, trait interactions

## Abstract

Under nitrogen limitation, plants increase resource allocation to root growth relative to shoot growth. The utility of various root architectural and anatomical phenotypes for nitrogen acquisition are not well understood. Nodal root number and root cross‐sectional area were evaluated in maize in field and greenhouse environments. Nodal root number and root cross‐sectional area were inversely correlated under both high and low nitrogen conditions. Attenuated emergence of root nodes, as opposed to differences in the number of axial roots per node, was associated with substantially reduced root number. Greater root cross‐sectional area was associated with a greater stele area and number of cortical cell files. Genotypes that produced few, thick nodal roots rather than many, thin nodal roots had deeper rooting and better shoot growth in low nitrogen environments. Fewer nodal roots offset the respiratory and nitrogen costs of thicker diameter roots, since total nodal root respiration and nitrogen content was similar for genotypes with many, thin and few, thick nodal roots. We propose that few, thick nodal roots may enable greater capture of deep soil nitrogen and improve plant performance under nitrogen stress. Synergistic interactions between an architectural and anatomical trait may be an important strategy for nitrogen acquisition. Understanding trait interactions among different root nodes has important implications in for improving crop nutrient uptake and stress tolerance.

**Abbreviations**
IBM: intermated B73 x Mo17
N: nitrogen
RIL: recombinant inbred line

## 1 INTRODUCTION

Developing stress‐tolerant, resource‐efficient crops is a key strategy for addressing the challenges of climate change, global food security, and land degradation (Blum & Jordan, 1985; Hunter et al., 2017; Mickelbart et al., 2015). Maize is a critical global crop, cultivated for food, fuel, and industrial uses (FAO, 2018). In intensive agriculture systems, nitrogen (N) fertilizers are over‐applied to maximize grain yield, yet over half of the applied nitrate leaches beyond the root zone and pollutes waterways, or is volatilized as greenhouse gases (Dhital & Raun, 2016; Hirel et al., 2011). In low‐input subsistence agriculture, which sustains half of the global population, maize is grown on marginal soils where nitrogen availability is a primary constraint to yield. Breeding nitrogen‐efficient and nitrogen‐stress tolerant maize varieties would therefore have substantial economic and environmental benefits.

Root systems have evolved structural and physiological strategies to forage for resources in complex, heterogeneous soil environments (Lynch, 2015; Rangarajan et al., 2018; York et al., 2013). When nitrogen is limiting, plants preferentially allocate resources to root construction and maintenance, as opposed to shoot growth (Bloom et al., 1985; Brouwer, 1962). However, the utility of investing in diverse root strategies is difficult to assess. For example, maize develops spatiotemporally and genetically distinct classes of embryonic and post‐embryonic roots, each composed of specialized axial (supportive, conducting) and lateral (branching, absorptive) roots (Demotes‐Mainard & Pellerin, 1992; Hochholdinger et al., 2004; Hoppe et al., 1986). Nodal roots emerge acropetally in whorls through development either belowground (“crown”) or aboveground (“brace”) (Hochholdinger et al., 2004). These nodal roots comprise the bulk of the axial root system, and, with their lateral roots, are responsible for the majority of water and nutrient uptake (Schneider et al., 2020). In addition, nodal root position influences size‐related traits, like diameter and number, which increase with younger node positions (i.e., nodes of a younger ontogenetic age) (York & Lynch, 2015). Although it is known that roots of a larger diameter are associated with younger nodal positions, there is still substantial genetic variation for root diameter and number of roots at these nodal positions. The understanding and development of a phene (“phene” is to “phenotype” as “gene” is to “genotype”) (York et al., 2013) for crop improvement is challenging and requires understanding of the fitness landscape, which for roots is largely unknown and likely to be highly complex. Understanding the utility of root phenes in variable and dynamic nitrogen regimes, phene trade‐offs for contrasting soil resources, and phene interactions may facilitate the incorporation of root phenotypes in crop breeding (Lynch, 2015, 2018).

Several root ideotypes, or integrated root phenotypes defined as breeding targets, have been proposed for improving nitrogen acquisition efficiency (York et al., 2013); for root system ideotypes, see Mi et al., 2010; White et al., 2013; Lynch, 2013, 2015, 2018; Lynch & Wojciechowski, 2015; Schmidt & Gaudin, 2017. To understand how different root phenes contribute to nitrogen stress adaptation, whole root system responses to nitrogen stress, as well as the utility of individual phenes, have been explored. Phene states such as steep crown root angle, fewer nodal roots, increased root cortical aerenchyma, and reduced lateral root branching have been associated with rapid, deep rooting and better yield under nitrogen stress among recombinant inbred lines (RILs) (Lynch & Wojciechowski, 2015; Saengwilai, Nord, et al., 2014; Saengwilai, Tian, et al., 2014; Trachsel et al., 2013; Zhan & Lynch, 2015), although modeling results suggest that phenes which maximize deep rooting may only provide benefits under precipitation regimes and soil textures that facilitate nitrate leaching (Dathe et al., 2016). Under low nitrogen conditions, some maize genotypes reduced the number and diameter of nodal roots, developed steeper root angles, increased the ratio of lateral to nodal root length, and increased the expression of nitrate transporters, among other changes (Gao et al., 2015; Gaudin et al., 2011; Gaudin McClymont & Raizada, 2011; Trachsel et al., 2013). Over time, maize breeding has indirectly resulted in increasing nitrogen use efficiency (Ciampitti & Vyn, 2012; DeBruin et al., 2017; York et al., 2015).

A comparison of commercial maize hybrids over the last century demonstrated that the most recent varieties had multiple changes in root phenotypes, including fewer (per node) but shallower nodal roots, delayed lateral root branching, and increased metaxylem vessel number and smaller diameter vessels. The functional‐structural model *SimRoot* demonstrated that a reduction in nodal root number makes century‐old genotypes as productive as modern root phenotypes in modern production environments (York et al., 2015). However, the utility of root phenes varies with nutrient regimes and depends on their fitness landscape, including its interactions with other phenes.

Understanding and accounting for phene interactions is important in determining the utility of a phene state for resource capture. Phenes that are beneficial to soil resource acquisition but have a high metabolic cost may have important interactions with root phenes that reduce the metabolic cost of resource capture. The formation of root cortical aerenchyma reduces the metabolic cost of the root and enables more carbon and nitrogen resources to be allocated to root growth (or formation of more nodal roots) for increased capture of nitrogen (Saengwilai, Nord, et al., 2014; Saengwilai, Tian, et al., 2014). For example, increased root cortical aerenchyma formation interacts with the production of more nodal roots, increasing plant growth in nitrogen‐limiting conditions up to 130% more than the expected additive effects (York et al., 2013). In common bean, root hair length and basal root growth angle interact in low phosphorus conditions, by affecting the placement of root resources in specific soil domains and increased plant growth up to twice the expected additive effect (Miguel et al., 2015). In common bean, simulations demonstrated that a greater number of basal root whorls and greater lateral root branching density were optimal in low phosphorus environments because more roots developed in the topsoil, which has greater phosphorus availability. In contrast, fewer basal root whorls and decreased lateral root branching enable deeper root growth and better nitrate capture (Rangarajan et al., 2018). A steep growth angle increases nitrogen capture in environments where nitrogen is available deep in the soil profile (Dathe et al. 2016; Trachsel et al. 2013). In silico, root hair length, density, geometry, and initiation show substantial synergism for P capture in Arabidopsis (Ma et al., 2001). Phene synergisms show strong interactions with environmental conditions, therefore may be important drivers in evolution and should be considered in crop breeding.

To understand the effects of combining potentially adaptive root traits, we evaluated the relationship of two root phenotypes—the number of nodal roots (a combination of the number of developed root nodes and the number of roots per node), and nodal root diameter (measured as root cross‐sectional area). While fewer nodal roots have been shown to improve nitrogen stress tolerance, the utility of anatomical traits, such as root cross‐sectional area, within the context of different nodal root number has not been studied. Previous work has suggested a benefit of maintaining larger root cross‐sectional area under low nitrogen (Yang et al., 2019), despite increased resource costs. However, interactions of a larger root cross‐sectional area with other root traits, such as a simultaneous reduction in nodal root number, could potentially offset resource costs (e.g., nitrogen and carbon) and be beneficial to nitrogen acquisition. For example, anatomical phenes that influence root cross‐sectional area are strong predictors of penetration strength of hard soils (Chimungu et al., 2015), root longevity, and resilience (Eissenstat et al., 2000), and may enable greater hydraulic conductance. Here we investigate a novel interaction between an architectural trait, fewer nodal roots, and an anatomical trait, a reduced cross‐sectional area, for enhanced nitrogen acquisition. We hypothesize that (a) genotypes with attenuated emergence of root nodes produce fewer, thicker nodal roots and (b) fewer nodal roots offset the respiratory and nitrogen costs of thicker diameter roots, resulting in greater plant performance and root depth under nitrogen stress. Maize recombinant inbred lines (RILs) contrasting in both nodal root number and root cross‐sectional area were used to evaluate combined trait effects on root respiration, nitrogen content, root length, root depth distributions, and shoot growth, in greenhouse and field experiments under high and low nitrogen conditions.

### 1.1 MATERIALS AND METHODS

#### 1.1.1 Plant material and growth conditions

A greenhouse experiment (eight RILs) and a field experiment (11 RILs) were performed in 2015. Experiments were performed using a subset of RILs from the IBM population. Maize seeds were provided by Dr. Shawn Kaeppler from the University of Wisconsin. Genotypes from all greenhouse and field studies are listed in Table S1.

Greenhouse mesocosm experiments were conducted in the greenhouse at University Park, PA (40° 45′ 36.0″ N, 73° 59′ 2.4″ W), with 14 hr photoperiod, maintained at approximately 28°C/26°C day/night, 40% RH, PPFD of 500 μmol/m^2^ s^1^ at the sixth leaf (Growmaster Procom, Micro Grow, Temecula, CA, USA). Germination and harvesting dates are listed in Table S1. Seeds were surface sterilized with 25% (v/v) commercial bleach for 3 min, rinsed with distilled water, then soaked in the seed fungicide Captan (0.2 g/L) for at least 10 min. Seeds were placed 2.5 cm apart in a row between two sheets of heavy weight seed germination paper (Anchor Paper Co.), then rolled up and placed vertically in an imbibing solution of 0.5 mM CaSO_4_, and dark‐incubated at 28°C for 2 days. Representative seedlings from each genotype were transplanted at about 5 cm depth.

Plants were grown in four replications in individual mesocosms consisting of polyvinyl chloride cylinders with an inner diameter of 15.5 cm and height of 1.54 m and lined with transparent 6 mm high‐density polyethylene film to facilitate root sampling. Genotypes were grown in a two‐way factorial complete block design. Each mesocosm was filled with a 30 L mixture consisting of 50% commercial grade medium sand (Quikrete), 27% horticultural grade fine vermiculite (D3, Whittemore Companies Inc.), 18% field soil, and 5% horticultural grade super‐coarse perlite (Whittemore Companies Inc.), by volume. Soil was collected from the top 20 cm of low nitrogen fields (Hagerstown silt loam: fine, mixed, semi‐active, mesic Typic Hapludalf) maintained at the Russell E. Larson Agricultural Research Center at Rock Springs, PA, air‐dried, crushed, and sieved through a 4‐mm mesh. A thin surface layer of perlite was added to each mesocosm to help retain moisture and reduce compression of media.

Plants were fertigated with high (7,300 μm) or low (140 μm) N nutrient solutions using drip rings (formulae in Table S2). Nutrient solutions were adjusted to pH 6.0 using KOH pellets, and maintained at this pH with KOH or HCl as needed. A dilute micronutrient foliar spray was applied uniformly as needed. Each mesocosm was saturated with 2.5 L of nutrient solution 1 day prior to transplant, then fertigated 200 ml per mesocosm every other day.

The field trials were conducted in 0.4‐ha fields maintained with split high and low nitrogen treatments at The Pennsylvania State University's Russell Larson Research Farm (40°42′40.915″N, 77°, 57′11.120″W, which has Hagerstown silt loam soil (fine, mixed, semi‐active, mesic Typic Hapludalf). To generate low N conditions, approximately 84 metric tons per ha sawdust was tilled into the soil in 2012 and 2013. High nitrogen fields were fertilized with 157 kg N/ha urea (46‐0‐0). The low nitrogen treatment did not receive any nitrogen fertilizer. Seeds were planted in rows with 76 cm row spacing at a density of approximately 56,800 plants ha^−1^. Genotypes were planted in three‐row plots in four replications per genotype in a split‐plot randomized block design with 88 total plots. Plants were sampled from the middle row of each plot. Planting and sampling dates are listed in Table S1. The average percent reduction due to nitrogen stress in shoot biomass and yield from all experiments is listed in Table S3. The soil nitrate distribution by depth under high and low nitrogen treatments is shown in Figure S1.

### 1.2 Plant sampling and measurements

In greenhouse studies, shoots were removed, dried at approximately 70°C for 72 hr, and stem and leaves were weighed separately. Whole root systems in media were removed intact within polyethylene liner bags, and media were gently washed off with a hose. Nodal roots were counted, and lengths measured by node. Two representative root segments each were excised from the second, third, and fourth nodes and preserved in 75% ethanol for anatomical analysis. Root respiration rate was measured on three 2‐cm nodal root segments (4–6 cm from the stem base) from each of these nodes, using a LI‐COR 6400XT (LI‐COR Biosciences) fitted with a closed custom chamber within 3 min after harvest. A subset of these roots was dried at about 70°C for 72 hr, weighed to determine specific root length, manually ground, and a 2 mg subsample was analyzed for carbon and nitrogen content using a CHN elemental analyzer (2,400 CHNS/O Series II, PerkinElmer). Each 30 cm of the entire root system, beginning at the stem base, was collected and dried at approximately 70°C for 72 hr to obtain total root biomass. Dried leaves were ground and a 2 mg subsample was analyzed for total nitrogen content as above.

In field studies, three representative plants from each plot were excavated using a shovel (Trachsel et al., 2011). Root crowns were separated from the shoots, soaked in water with 0.5% v/v detergent (Liquinox, Alconox, Inc.) for approximately 10 min, and hosed to remove remaining soil. Each node of roots was excised, node 1 being defined as the coleoptilar node, and three representative roots from nodes 2, 3, and 4 in the greenhouse and nodes 1, 2, and 3 in the field were sampled at 2–4 cm from the base of the stem. Nodes were identified by dissecting from the youngest nodes to the oldest nodes. Anatomical samples were preserved in 75% ethanol for anatomical processing.

For a subset of plants, nodal roots (>2 cm) were counted by node and recorded as crown (emerging below the soil line and having no pigmentation) or brace roots (emerging above the soil line and having pigmentation). Shoot biomass was separated into stem, leaves, and ears, dried at approximately 70°C for 72 hr, and weighed. Dried leaves were ground and analyzed for total nitrogen content with a CHN elemental analyzer. Ears from five plants per plot were collected at physiological maturity, dried to approximately 15% moisture content, shelled and weighed.

To determine relative root lengths and depths, soil cores 60 cm in depth and 5 cm in diameter were taken manually with a sledgehammer using a steel coring tube and plastic liner (Giddings Machine Co.), between two plants in each plot. Soil cores were separated into 10 cm segments and roots were extracted using a custom root washer. Roots were placed in water on a clear plastic tray, scanned (Epson Perfection V700 Photo, Epson America, Inc.) at 600 dpi, and analyzed for root length separated by diameter classes (e.g., 0.2, 0.5, 1 mm) to estimate lateral versus nodal (i.e., axial) root lengths using Winrhizo Pro software (Regent Instruments).

In greenhouse experiments, medium samples were collected at different depths in the mesocosm, air‐dried, homogenized, and samples of equal mass were tested for nitrate content was using a LAQUA nitrate meter (Spectrum Technologies) according to the manufacturer's instructions. For field experiments, soil cores were taken, separated by depth, homogenized, dried at 70°C for 72 hr, and extracted as above. The low nitrogen treatment had on average 36% reduced soil nitrogen in the greenhouse and 36% reduced soil nitrogen in the field compared to the high nitrogen treatment (Figure S1).

The nitrogen treatment was confirmed in all experiments by a ~46% vegetative biomass growth reduction in greenhouse experiments, a ~30% vegetative biomass growth reduction in field experiments, and a ~20% yield reduction in field experiments in nitrogen stress compared to control conditions (Table S3).

### 1.3 Image and statistical analysis

For field experiments, the middle portion of two representative root segments per node of each plant was ablated and imaged using laser ablation tomography (LAT) (Hall et al., 2019; Strock et al., 2019). In brief, the root is moved into the laser beam of a nanosecond pulsed UV laser (Avia 355‐7000, Coherent) at about 30 μm/s and as each surface is ablated and exposed, images are captured. Image scale was 1.173 pixels per micron. Select greenhouse‐grown root segments required preprocessing in a critical point dryer (Leica EM CPD300, Leica Microsystems) to prevent sample desiccation during laser ablation. Roots were placed in histo prep tissue capsules (Fisherbrand, Fisher Scientific) and gradually dehydrated 75%–100% ethanol prior to critical point drying.

For greenhouse experiments, two ethanol‐preserved roots from each node (2, 3, and 4) were manually sectioned using fresh double‐edged razor blades, wet‐mounted, and visualized using a Diaphot inverted light microscope (Nikon Inc.) under 4X magnification with a mounted CCD camera (Nikon DS‐Fi1 camera with DS‐U2 USB controller, Nikon, Inc.). Images were captured using NIS Elements F 4.30.00 software (Nikon, Inc.) at a scale of 390.7 pixels per mm, using 1,280 × 920 pixel resolution. Two representative cross‐sections images per root were selected for analysis.

Images were analyzed using custom macros created with the open‐source ObjectJ plug‐in in ImageJ (Rasband, 2015) in which cortex, stele, aerenchyma, vessel, and cell outlines were manually traced, and cell files manually counted (Figure S2). This allowed careful quantification of cell and vessel sizes. A total of 17 anatomical phenes were measured on each root cross‐sectional image (Figure S2).

Statistical analysis and visualizations were generated using R version 3.3.1(R Core Team, 2018). Data were analyzed by linear regression, Pearson correlation coefficients, and Tukey's HSD (honest significant difference). Few, thick phenotypes are classified as having a root cross‐sectional area greater than 2 µm and a nodal root number less than 15. Many, thin phenotypes are classified as having a root cross‐sectional area less than 1 µm and a nodal root number greater than 30. Phenotypes not meeting these criteria were excluded from these analyses. Bar plots were generated using data aggregation functions from the package *plyr* (1.8.6) and plotting functions from the package *ggplot2* (3.0.0) (Wickham, 2016).

## 2 RESULTS

### 2.1 Reduced nodal root number was associated with increased cross‐sectional area among maize RILs

**FIGURE 1:**
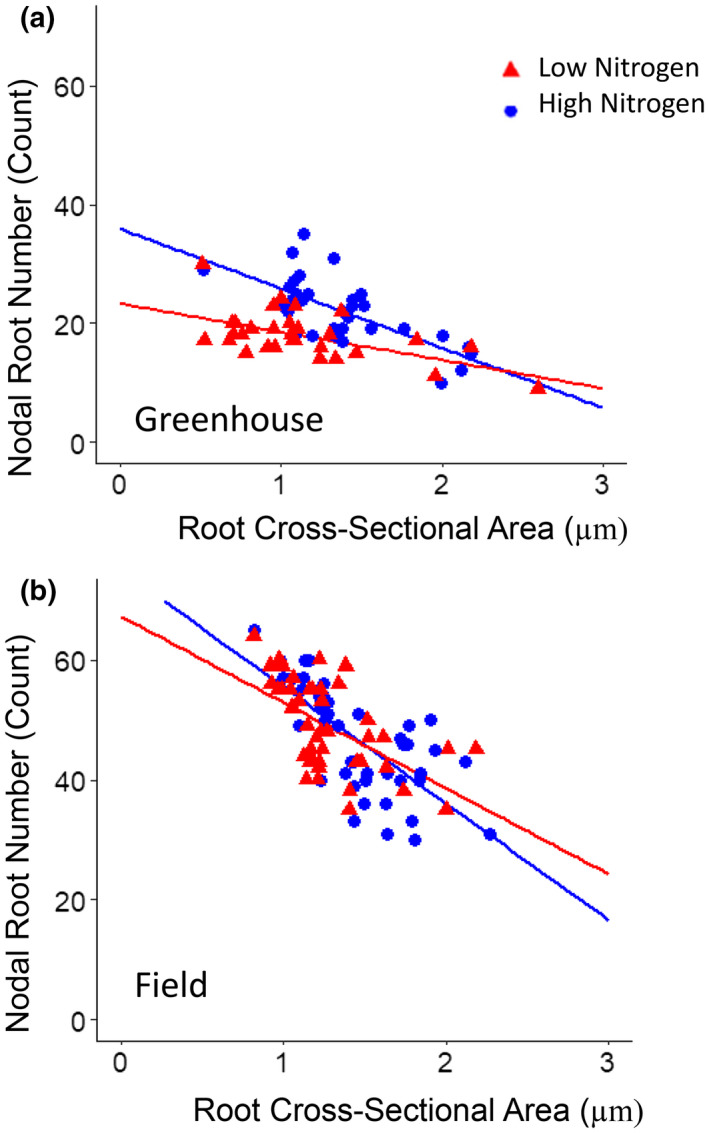
Relationship between nodal root number and root cross‐sectional area among maize RILs. Linear regression of number of nodal roots emerged at sampling and root cross‐sectional area averaged from two second and third node roots, from individual plants of a subset of IBM RILs grown in high (HN, blue) or low nitrogen (LN, red) treatments in the: (a) greenhouse (HN: *R* ^2^ = .48, *p* = .003; LN: *R* ^2^ = .3, *p* = .004) and (b) field (HN: *R* ^2^ = .5, *p* = .003; LN: *R* ^2^ = .32, *p* =.002)

**FIGURE 2:**
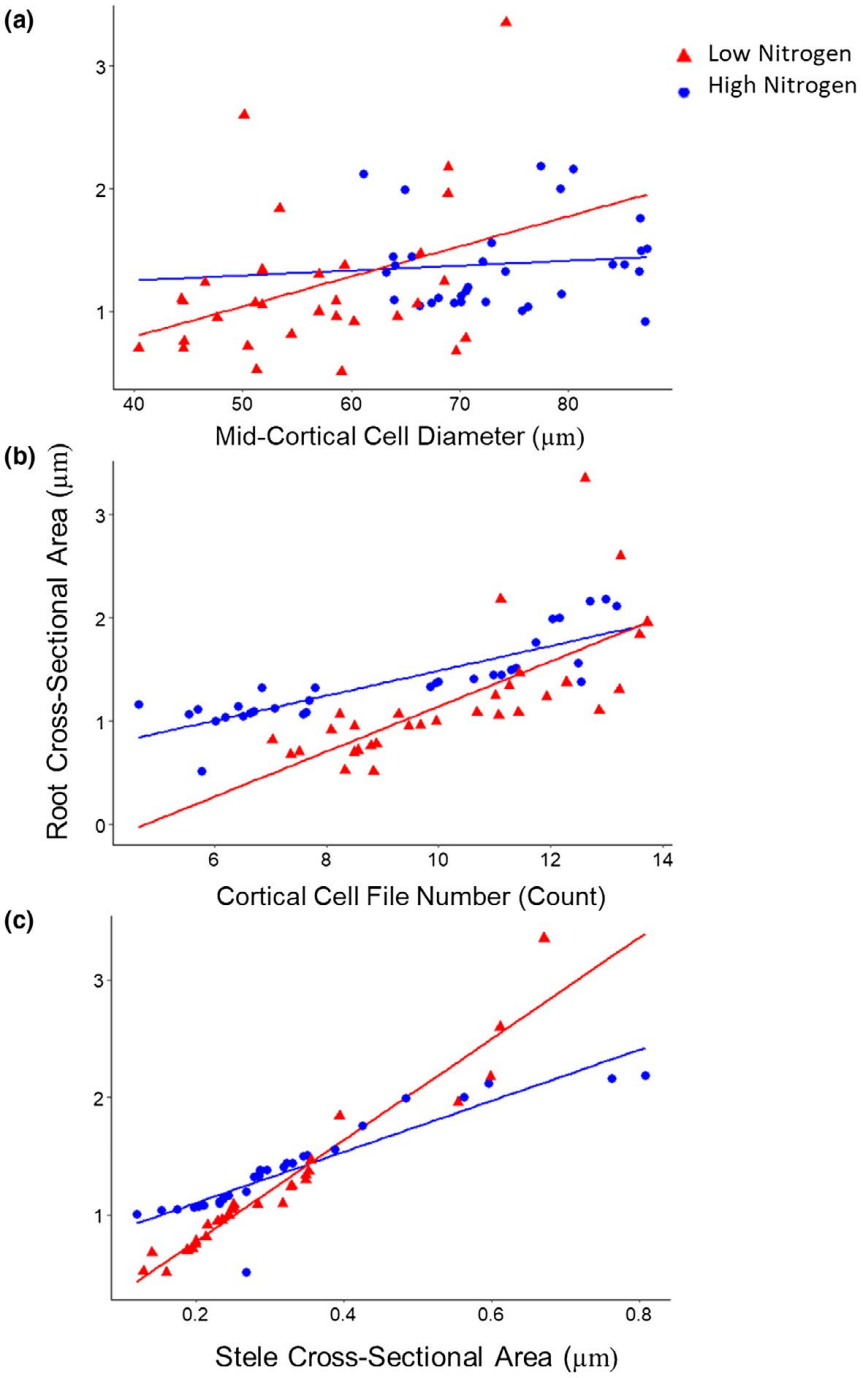
Relationship between nodal anatomical traits and root cross‐sectional area among a subset of maize IBM RILs. Linear regression of the following nodal anatomical traits against root cross‐sectional area averaged from two second and third node roots (fully developed in all genotypes at sampling), from individual plants of a subset of maize IBM RILs grown in high (HN, blue) or low nitrogen (LN, red) treatments in the greenhouse: (a) mid‐cortical cell diameter (HN: *R* ^2^ = .001, *p* = .004; LN: *R* ^2^ = .13, *p* = .001), (b) cortical cell file number (HN: *R* ^2^ = .72, *p* = .004; LN: *R* ^2^ = .5, *p* = .003), and (c) stele cross‐sectional area (HN: *R* ^2^ = .081, *p* = .003; LN: *R* ^2^ = .93, *p* = .002)

Genotypes with a greater nodal root cross‐sectional area, averaged from nodal roots in the second and third nodes, had a reduced number of nodal roots among RILs in high and low nitrogen conditions in the greenhouse and field (Figure 1a,b). Differences in nodal root number were driven by the number of root nodes, rather than nodal occupancy (Figure S3). Contrasts in root cross‐sectional area among maize RILs were most strongly related to cortical cell file number and stele area, rather than cortical cell diameter (Figure 2). Cortical cell diameter was strongly positively correlated with root cross‐sectional area under low nitrogen, but not high nitrogen conditions (Figure 2a).

### 2.2 Fewer, thicker nodal roots were associated with better shoot growth under nitrogen stress in maize RILs

**FIGURE 3:**
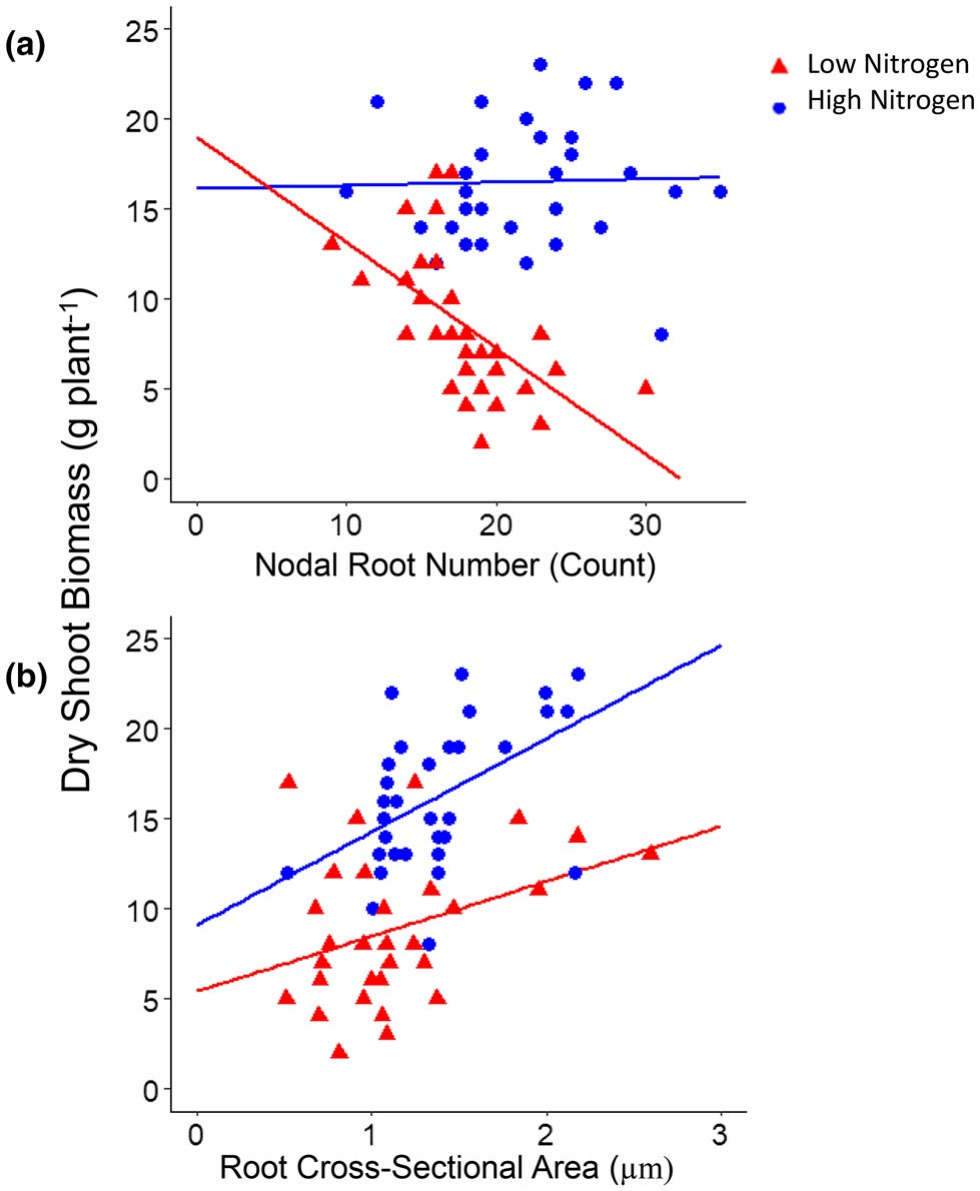
Relationship between shoot biomass, nodal root number, and root cross‐sectional area in a subset of maize IBM RILs. Linear regression of (a) total number of nodal roots at sampling (HN: *R* ^2^ = .11, *p* = NS; LN: *R* ^2^ = .35, *p* = .003) and (b) root cross‐sectional area (HN: *R* ^2^ = .25, *p* = .003; LN: *R* ^2^ = .19, *p* = .002) averaged from two second and third node roots, against total dry shoot biomass, from individual plants of a subset of maize IBM RILs grown in high (HN, blue) or moderate low nitrogen (LN, red) treatments in the greenhouse

**FIGURE 4:**
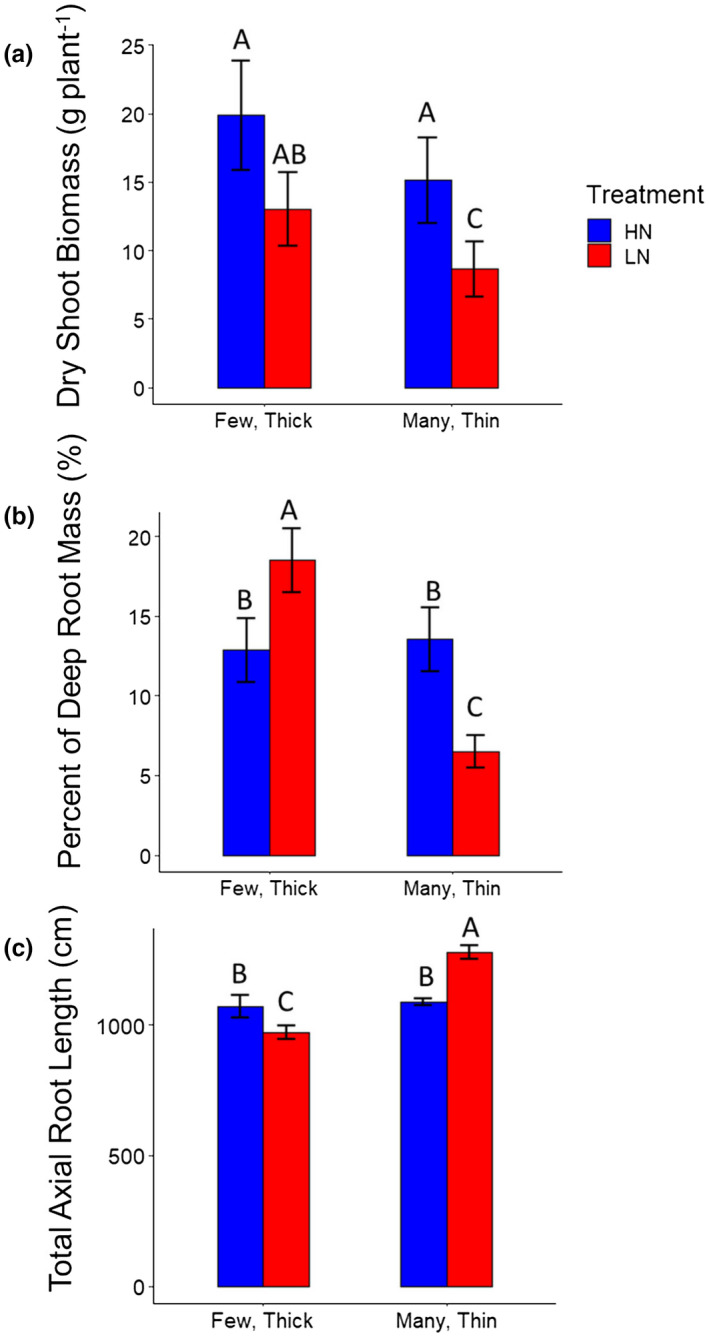
Relationship between shoot biomass, total axial root length, deep root mass, and root phenotypes among maize RILs. (a) Lines with few, thick nodal roots have greater dry shoot biomass in low nitrogen compared to lines with many, thin nodal roots. (b) Lines with few, thick nodal roots have greater deep root mass in low nitrogen conditions compared to lines with many, thin nodal roots. (c) Lines with few, thick nodal roots have a reduced total axial root length when compared to lines with many, thin nodal roots. Few, thick phenotypes are classified as having a root cross‐sectional area greater than 2 μm and a nodal root number less than 15. Many, thin phenotypes are classified as having a root cross‐sectional area less than 1 μm and a nodal root number greater than 30. Phenotypes not meeting this criteria were excluded from these analyses. Data shown are means ± standard error (SE) for three genotypes per group averaged from second and third nodal roots (*n* = 24). Means with the same letters are not significantly different (*p* ≤ .05) according to Tukey's HSD

Fewer nodal roots were correlated with greater shoot mass under nitrogen stress (Figure 3a). In contrast, there was no significant relationship between nodal root number and dry shoot biomass under high nitrogen in these studies (Figure 3a). Larger root cross‐sectional area was positively correlated with shoot biomass under low and high nitrogen conditions (Figure 3b). Lines with the few, thick nodal root phenotype had significantly greater dry shoot biomass in low nitrogen conditions when compared to lines with the many, thin nodal root phenotype (Figure 4a).

### 2.3 Genotypes with fewer, thicker nodal roots produced less total nodal root length but deeper roots

**FIGURE 5:**
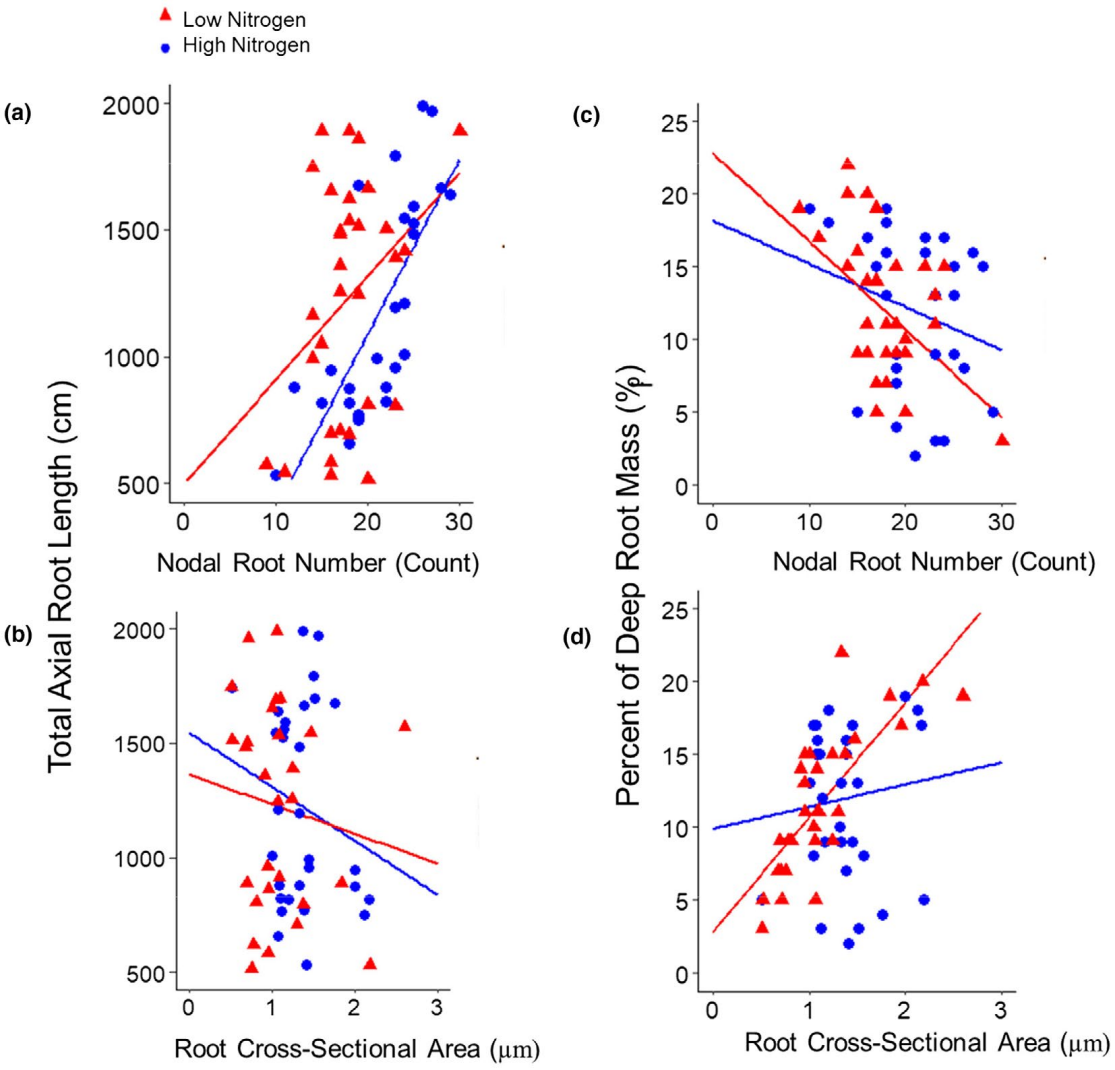
Relationship between total axial root length, deep root mass, root number, and cross‐sectional area among a subset of maize RILs. Linear regression of (a) total number of nodal roots sampled (HN: *R* ^2^ = .03, *p* = NS; LN: *R* ^2^ = .23, *p* = .03) at harvest against the percent of total root biomass below 124 cm and (b) root cross‐sectional area sampled (HN: *R* ^2^ = .013, *p* = NS; LN: *R* ^2^ = .57, *p* = .004) averaged from two second and third node roots and (c) total number of nodal roots sampled (HN: *R* ^2^ = .05, *p* = NS; LN: *R* ^2^ = .06, *p* = .005) at harvest and (d) root cross‐sectional area sampled (HN: *R* ^2^ = .05, *p* = NS; LN: *R* ^2^ = .07, *p* = .005) averaged from two second and third node roots, against total axial root length summed from node 2 through all developed root nodes, from individual plants of eight maize IBM RILs grown in high (HN, blue) or moderate low nitrogen (LN, red) treatments in the greenhouse. Plants with missing values were excluded

**FIGURE 6:**
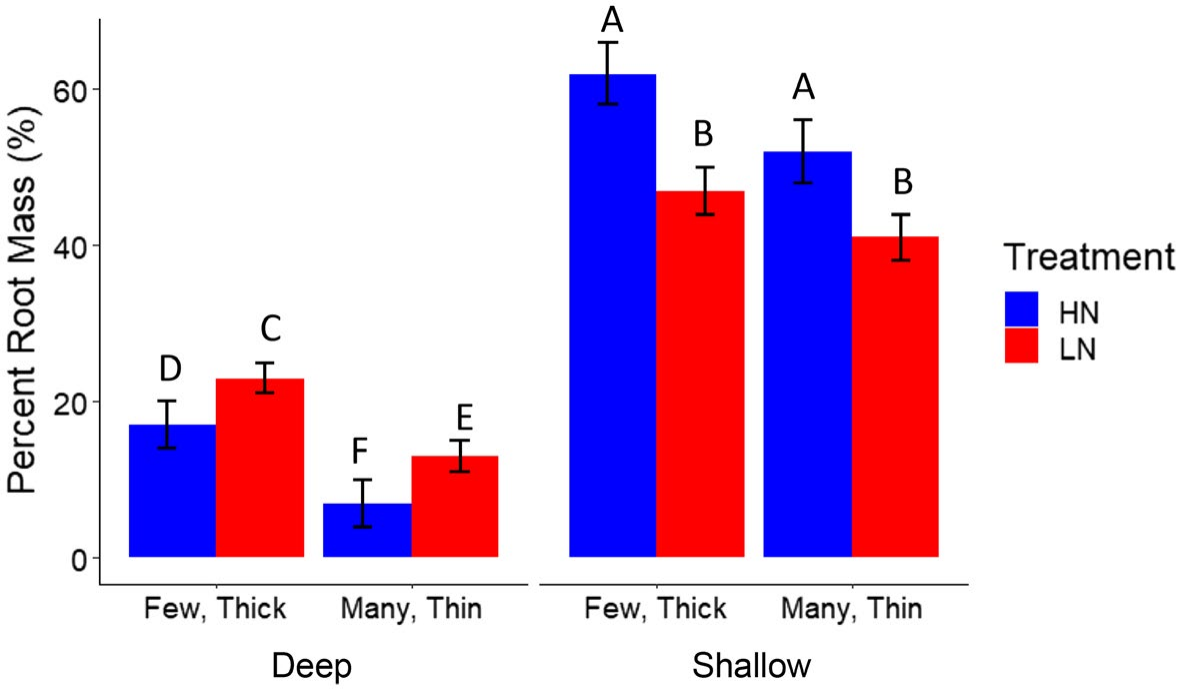
Relative root depth distributions among maize IBM RILs. Means ± SE of the percent of total root mass located below 124 cm (deep) percent of total root mass in the top 30 cm of media (shallow) among a subset of IBM RILs in the greenhouse. High and low nitrogen treatments are indicated (HN, blue; LN, red). Few, thick phenotypes are classified as having a root cross‐sectional area greater than 2 μm and a nodal root number less than 15. Many, thin phenotypes are classified as having a root cross‐sectional area less than 1 μm and a nodal root number greater than 30. Phenotypes not meeting these criteria were excluded from these analyses. Means with the same letters are not significantly different (*p* ≤ .05) according to Tukey's HSD

The total axial root length produced (the product of nodal number and the average root length) was most strongly related to nodal root number under high and low nitrogen conditions, whereas root cross‐sectional area was only weakly negatively correlated with total root length under low nitrogen (Figure 5a,b). Lines with the few, thick phenotype had significantly reduced total axial root length in low nitrogen conditions when compared to lines with many, thin nodal roots (Figure 4c). Larger root cross‐sectional area and fewer nodal roots were significantly correlated with deeper relative root distribution among RILs in low nitrogen conditions (Figures 4b and 5c,d). Lines with the few, thick nodal root phenotype had significantly greater deep root mass in low nitrogen conditions when compared to lines with the many, thin nodal root phenotype (Figure 4b). Under nitrogen stress, a greater proportion of the root system became deeply distributed. The percent of roots in the shallowest 20 cm decreased, while the percent of roots in the deepest layers increased (Figure 6).

### 2.4 Nitrogen stress effects on root system phenotypes were node‐specific

In greenhouse‐grown plants, nitrogen stress reduced average nodal root length, root cross‐sectional area, and nodal occupancy overall, with effects differing by node (Figure S4a–c). In high nitrogen conditions, axial root length decreased in each node; roots from the first two nodes typically reached the bottom of the mesocosm (150 cm) by sampling, 5 weeks after germination (Figure S4a). Root cross‐sectional area increased with each younger root node across genotypes, and nodal occupancy increased from the third node onward. In low nitrogen, fewer plants, particularly plants that develop fewer nodal roots, had developed a sixth node resulting in an apparent increase in nodal occupancy in node 6 (two plants measured in high nitrogen, one plant measured in low nitrogen) (Figure S4b,c).

### 2.5 Fewer nodal roots offset increased carbon and nitrogen costs of thicker nodal roots

**FIGURE 7:**
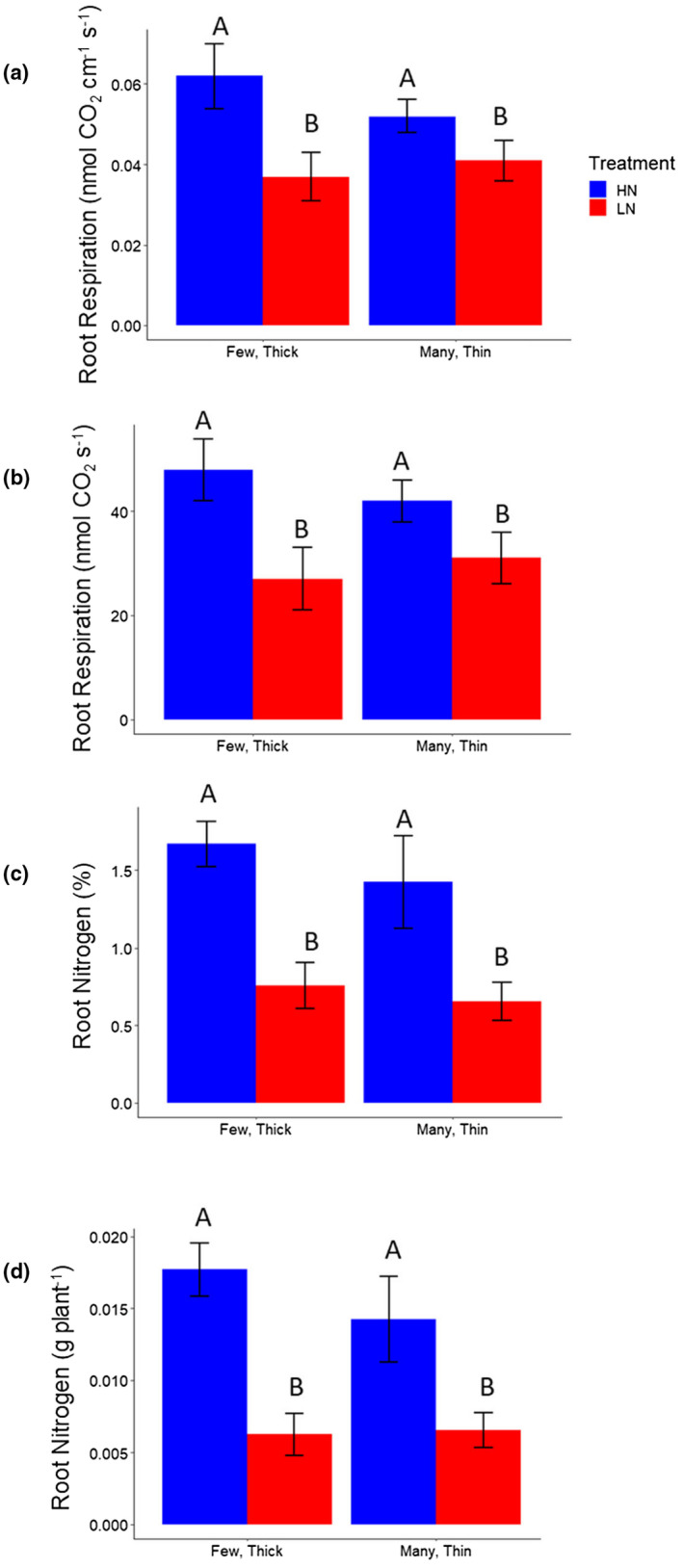
Root respiration and nitrogen content among a subset IBM RILs. Means ± SE of (a) root respiration rates, averaged from six axial roots from each genotype, 2–5 cm from stem base, from nodes 2 and 3, and (b) total root respiration (estimated as root respiration rate multiplied by total axial root length in the given node) of nodes 2 and 3, in the greenhouse. Means ± SE of (c) root nitrogen content (percent by mass) from the top 30 cm of the root system (all root classes, homogenized), evaluated in a subset of replicates, and (d) total root nitrogen in the top 30 cm of the root system (percent root nitrogen multiplied by dry root mass in the top 30 cm), in the greenhouse. High and low nitrogen are indicated (HN, blue; LN, red). Few, thick phenotypes are classified as having a root cross‐sectional area greater than 2 μm and a nodal root number less than 15. Many, thin phenotypes are classified as having a root cross‐sectional area less than 1 μm and a nodal root number greater than 30. Phenotypes not meeting these criteria were excluded from these analyses

Nitrogen stress significantly decreased nodal root respiration per unit of root length, and in combination with reductions in nodal root length, resulted in substantial reductions in total nodal root respiration per plant (Figure 7a,b). Similarly, reduced root nitrogen content combined with decreased root biomass resulted in substantial reduction in the percent root nitrogen and total root nitrogen (grams per plant) across genotypes (Figure 7c,d). Root respiration per unit of root length was significantly positively related to root cross‐sectional area in high and low nitrogen conditions (Figure S5). However, when multiplied by the total number and length of nodal roots, total nodal root respiration was similar for genotypes with many, thin and few, thick nodal roots (Figure 7a,b). Total root nitrogen content also did not significantly differ between phenotypes when multiplied by root mass indicating that fewer nodal roots offset increased carbon and nitrogen costs of thicker nodal roots (Figure 7b,d). Total root nitrogen was similar across genotypes under nitrogen stress (Figure 7d).

## 3 DISCUSSION

This study explored the synergistic interaction of root anatomical and architectural traits for nitrogen capture in maize, and identified phenotypes associated with improved plant performance in nitrogen stress: a reduced number of developed root nodes (and thus total number of nodal roots emerged in a given period) and increased nodal root cross‐sectional area. Previously, the utility of reduced nodal root number was demonstrated to have utility for enhanced nitrogen capture (Saengwilai, Tian, et al., 2014). However, here we identified a previously uncharacterized interaction between nodal root number and cross‐sectional area to further improve nitrogen acquisition and nitrogen stress tolerance. Genotypic contrasts in root cross‐sectional area were driven primarily by differences in cortical cell file number and stele area, with some differences in cortical cell diameter under low nitrogen (Figure 2). Genotypes with fewer, thicker roots developed root nodes more slowly, produced less total nodal root length, and invested more carbon and nitrogen per unit of root length (Figures 4, 5, 6). The phenotype of fewer, thicker nodal roots was associated with deeper root distribution and resulted in greater shoot growth under nitrogen stress.

The number of crown roots is an important determinant of soil resource capture (Lynch, 2013) and large genetic variation is present for this trait in maize (Gao & Lynch, 2016; Gaudin, McClymont, Holmes, et al., 2011; Guo et al., 2019; Saengwilai, Tian, et al., 2014; Sun et al., 2018; Trachsel et al., 2011). Among field‐ and greenhouse‐grown lines, there was a significant, negative correlation between nodal root number and shoot mass under nitrogen stress (average of 50% biomass reduction) (Figure 3). Fewer nodal roots is advantageous for the capture of mobile nutrients like nitrogen (Saengwilai, Tian, et al., 2014) and water (Gao & Lynch, 2016), while a larger number of crown roots is advantageous for the capture of phosphorus (Sun et al., 2018) and presumably other immobile nutrients (Lynch, 2019). In low nitrogen conditions, fewer nodal roots is advantageous as more resources will be available for the development of longer, deeper roots resulting in greater nitrogen acquisition, growth, and yield when compared to genotypes with many crown roots. Functional‐structural modeling in *SimRoot* suggested that the effects of reduced nodal root number on nitrate uptake were similar regardless of whether this reduction came from delaying the emergence of roots (reduced time with a given number of roots), or producing fewer roots per node (York, 2014).

We observed a consistent positive correlation between root cross‐sectional area and shoot mass under high and low nitrogen, among greenhouse‐grown and field‐grown RILs, suggesting a potential benefit of thicker nodal roots (or a benefit of thicker roots given concurrent decrease in root number, or other unknown linked traits) for nitrogen stress tolerance. Roots with a smaller root cross‐sectional area would be expected to have enhanced performance due to a reduced metabolic cost. However, among RILs, fewer nodal roots reduced root carbon and nitrogen costs, offsetting the increased respiratory costs and nitrogen content of thicker root diameter. We propose that fewer, thicker nodal roots have utility in nitrogen stress. Fewer nodal roots offset resource costs of thicker roots while enabling greater hydraulic conductance and soil penetration strength which have utility for nitrogen capture.

Thicker root diameter has been associated with better performance in hybrids in nitrogen stress, partly due to a positive allometric relationship with plant size (see Yang et al., 2019). Increased Root cross‐sectional area has been associated with increased root penetration strength (Chimungu et al., 2015; Striker et al., 2007), increased hydraulic conductance (Jordan et al., 1993), and lodging resistance (Stamp & Kiel, 1992). Larger mid‐cortical cell diameters and fewer cortical cell files were associated with reduced metabolic costs per root length, deeper rooting, and enhanced drought tolerance in IBM RILs (Chimungu et al., 2014a, 2014b).

Seminal, primary, and lateral root classes were not evaluated in detail in this study. Decreases in lateral to nodal root length ratio have been suggested as a potential adaptation to nitrogen stress (Zhan & Lynch, 2015). Fewer lateral branches decreases intra‐ and inter‐root competition and decreases root respiration costs enabling deeper root growth and nitrogen capture (Zhan & Lynch, 2015). Additional research is needed to reveal whether plants with few, thick roots invested the additional resources in other root classes, such as additional lateral root proliferation, which could result in greater specific root length overall and increased capacity for nitrate uptake.

Nitrogen stress has large effects on root phenotypes. In low nitrogen, root growth angles became steeper (Trachsel et al., 2013), the proportion of root cortical aerenchyma increases (Gao et al., 2015; Saengwilai, Nord, et al., 2014), nodal root diameter increases (Yang et al., 2019), and in this study, total nodal root length is reduced (Figure 4) and is the number of nodal roots per node (Figure S3). As a result of changes in root number, diameter, angle, and length, in the current study, root systems became more deeply distributed under nitrogen stress in the field and greenhouse, in accordance with previous studies (Dathe et al., 2016; Gaudin, McClymont, Holmes, et al., 2011; Postma et al., 2014; Saengwilai, Tian, et al., 2014; Trachsel et al., 2013; Zhan & Lynch, 2015). The extent to which the nitrogen regime affected root depth distribution differed among genotypes, but all genotypes showed a substantial reduction in the proportion of root mass in the top 30 cm under low nitrogen in the greenhouse. Similarly, nitrogen stress induced a significant reduction in the percent of root length in the top 20 cm of soil in the field, and an increase in the percent of root length in the deepest 20 cm, among RILs. Finally, nitrogen stress induced a well‐established increase in root to shoot mass allocation, which can be observed generally as a shift in allometric scaling between root and shoot mass across genotypes. Shoot mass was also weakly correlated with root cross‐sectional area under high nitrogen in some experiments, suggesting that a positive allometric relationship of root traits with plant size may exist. The integration of root and shoot responses to nitrogen stress in maize, and the impacts of different anatomical, morphological, and architectural strategies on nitrogen uptake and utilization efficiency are complex and merit further research.

Nitrogen stress significantly decreased the number of roots per node in all nodes except the first two. However, genotypes varied in the node at which nodal occupancy began to decrease, and some genotypes (e.g., IBM181) showed no significant decrease in occupancy across nodes (Figure S3). Previous studies have shown that timing of the initiation of root node primordia is staggered, and all initiated primordia always elongate in the first five nodes (Aguirrezabal et al., 1993; Girardin et al., 1986; Sharman, 1942). Aguirrezabal et al. (1993) also suggested that in contrast to the first five nodes, subsequent nodes regularly initiated excess root primordia which did not elongate, increasing their sensitivity to carbon availability and the potential for plastic responses (Aguirrezabal et al., 1993). If root initiation preceded the onset of stress signaling, nodal occupancy would likely not be affected (e.g., Pellerin, 1994; in contrast to elongation rate, which could therefore be considered more “plastic”). Therefore, the timing and level of nitrogen stress could directly impact the potential for decreased nodal root number.

Our results demonstrate that few, thick nodal roots are beneficial to plant growth when compared to plants with many, thin nodal roots. Small differences in data trends between the field and greenhouse could be attributed to differences in the growth environment, the spatiotemporal location of nitrogen in the soil, and differences in soil bulk density. In both the greenhouse and field, we employed “near isophenic” (i.e., similar in all root traits except for crown root number and root cross‐sectional area) RILs to explore the physiological utility of root traits in nitrogen stress. RILs share a common genetic background (i.e., descending from the same two parents) and are suited to the physiological analysis of phenotypes controlled by multiple alleles. RILs are suitable for this study because they are closely related genotypes therefore minimizing the risk of effects from epistasis, genetic interactions, and pleiotropy which may cofound interpretation of results from other related lines (Zhu et al., 2006).

We highlight an integrated root phenotype related to a developmental process (the rate of root node development) which may underlie phenotypic contrasts and physiology. The integration of nodal root number and root anatomy may have important implications for soil resource capture. Root diameter is a strong predictor of penetration strength in hard soils (Chimungu et al., 2015), root longevity, and resilience (Eissenstat et al., 2000), and may enable greater hydraulic conductance. In addition, fewer nodal roots enable deeper rooting and potentially reducing the spatial overlap among axial roots and therefore reducing intra‐plant competition for soil resources. Understanding trait interactions among different root nodes has important implications in ideotype breeding of crop stress tolerance.

## CONFLICT OF INTEREST

The authors declare no conflict of interest.

## Supporting information

Fig S1‐S5

Table S1‐S3
