# Supplementary material for: Nodal root diameter and node number in maize (Zea mays L.) interact to influence plant growth under nitrogen stress
Source: Plant Direct. 2021 Mar 16;5(3):e00310. doi: 10.1002/pld3.310 (PMC7963125; doi:10.1002/pld3.310)
Supplement: Supplementary file 1 — Fig S1‐S5 [file PLD3-5-e00310-s001.pdf]

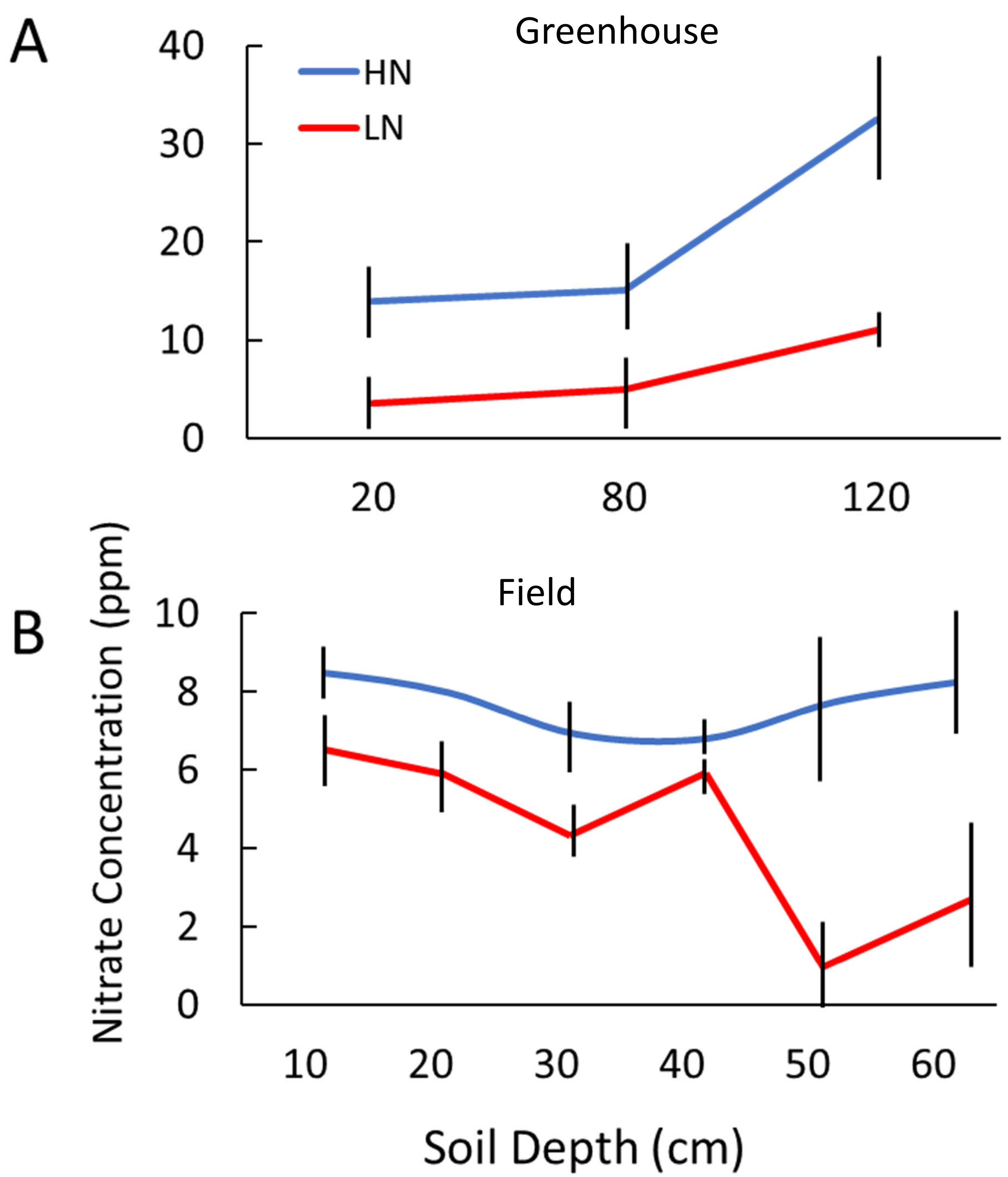

Fig S1. Nitrate concentrations by depth in greenhouse and field experiments. Nitrate concentration from soil or media extracts, averaged across soil or media samples from at least two replicates in the greenhouse and field from the indicated depths in high (HN, blue) or low (LN, red) nitrogen treatments. Error bars represent the standard error.

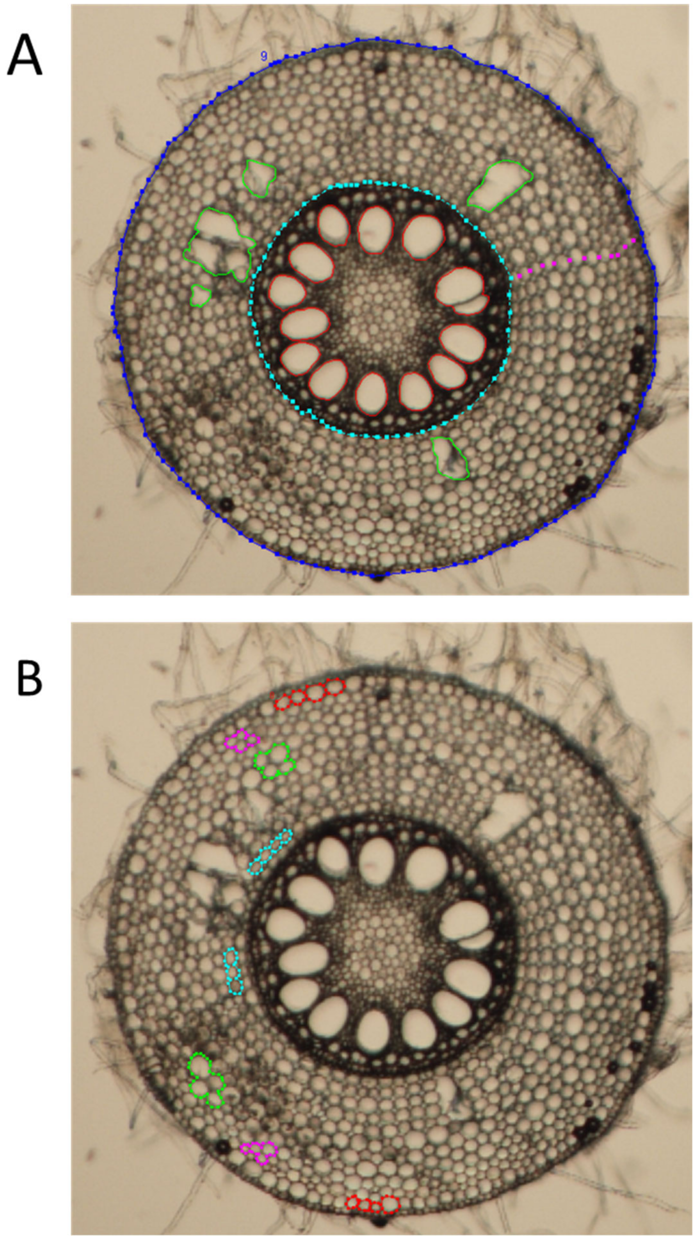

Fig S2. Maize root cross-section image analysis. An ObjectJ macro in ImageJ was created to semi-automate analysis of root-cross section images. The same anatomical traits were analyzed in LAT and manually sectioned roots, with minor differences in methodology. An example of an analyzed image from manual cross-sections. (A) The root (dark blue, outer), stele (cyan, inner), and aerenchyma (green) were outlined and total areas and ratios calculated. Individual metaxylem vessels were traced (red) and individual and total areas determined. Vessels beginning to divide were traced as a single vessel. Images were zoomed in to allow accurate tracing along the outer edge of each vessel. Cortical cell file number was manually counted in three positions to account for root asymmetry, and a representative axis across the cortex was selected to record a representative cell file count (the count of pink points) and measure cell diameters of each cell file (distance between every consecutive pink point). The innermost cell layer (i.e. distance between the innermost pink point and the stele boundary) was often incomplete and was not recorded. The cell diameters were used to calculate the cell sizes of the hypodermis (HYP), outermost (OUT) and innermost (INN). (B) For one full replicate, the inner (cyan), mid (green), outer (pink), and hypodermis (red) cortical cell sizes were determined and used to validate estimates from cell diameters as described above. Up to eight representative cells per layer were traced and average cell cross-sectional areas were calculated. Images were zoomed in to allow careful tracing of the outer edge of the cells; cell walls were included in the trace. Unlike LAT images, manual cross-sections from greenhouse-grown plants showed intact epidermis cells and root hairs.

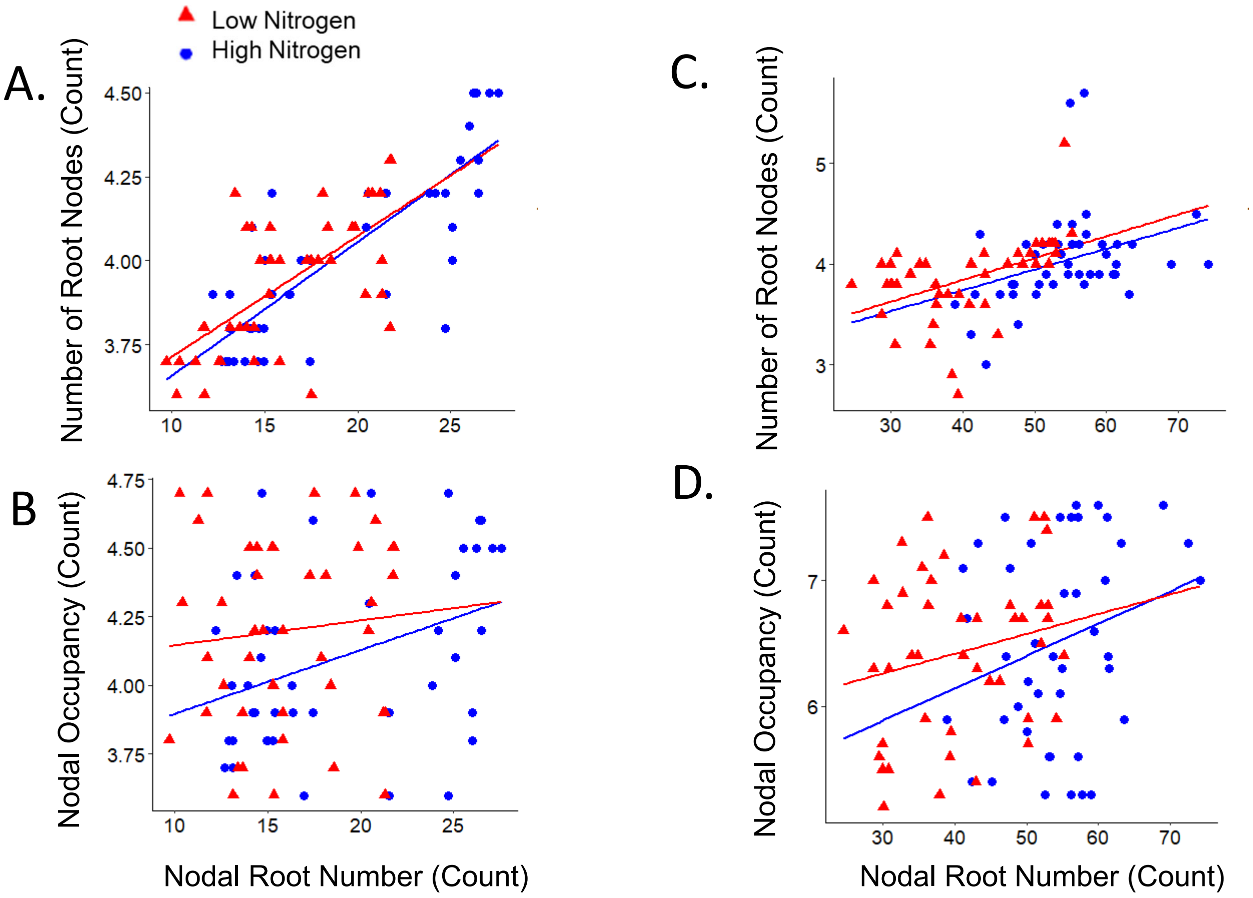

Fig S3. Genotypic contrast in nodal root number and diameter among maize RILs in the field and greenhouse. (A) total number of nodal roots emerged and number of root nodes at harvest in greenhouse mesocosms, (B) nodal root number and nodal occupancy from nodes 2, 3, and 4 in greenhouse mesocosms (C) total number of nodal roots emerged and number of root nodes at harvest in the field, (D) nodal root number and nodal occupancy from nodes 1, 2, and 3 in the field. Data points represent means of four replications for each genotype. High and low nitrogen treatments are indicated (HN, blue; LN, red).

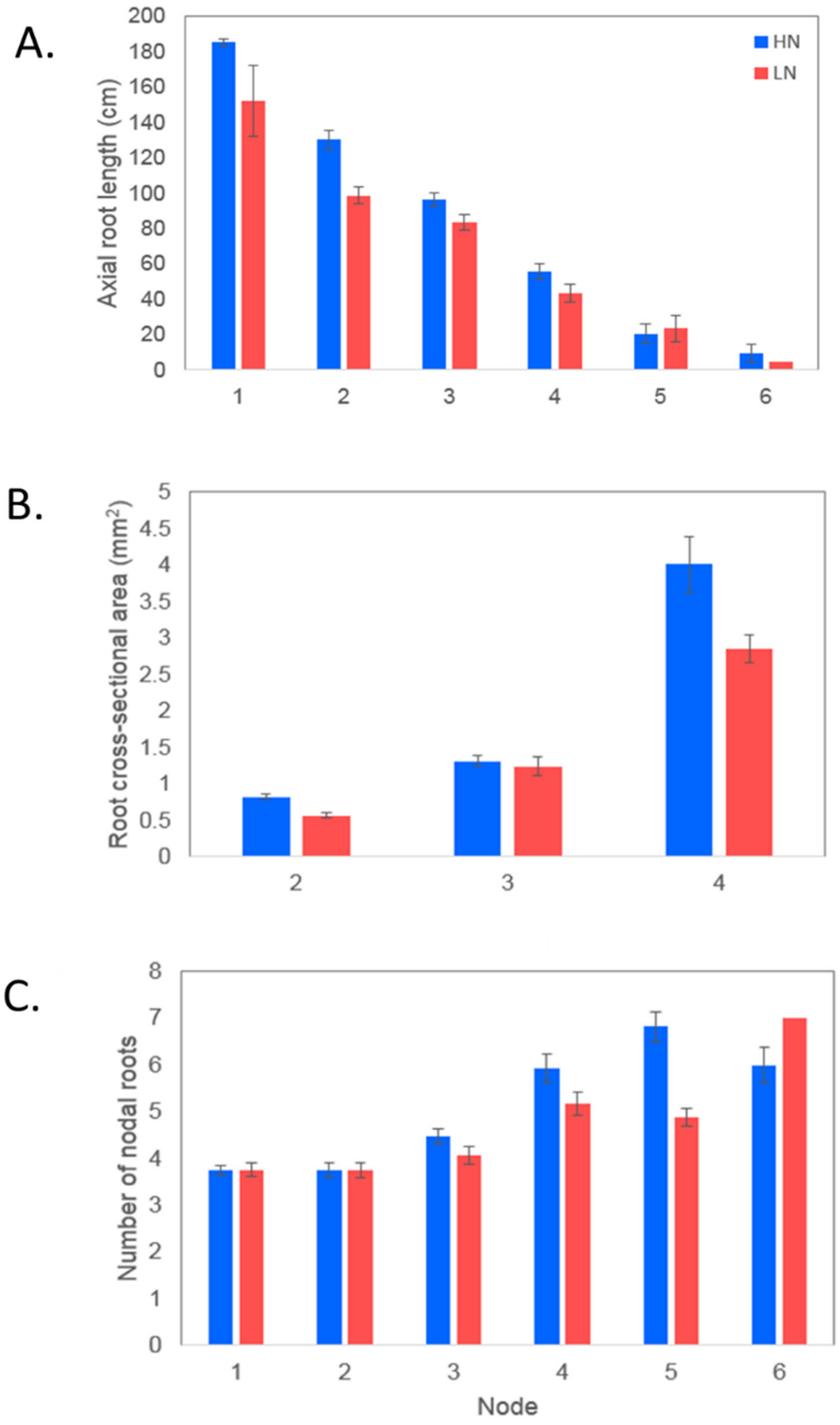

Figure S4. Axial root lengths, diameters, and occupancy by node under high and low nitrogen in IBM RILs. Means ± SE of (A) the average axial root length (ARL) by node at time of harvest, and (B) average RXA by node, and (C) average number of roots per node (NO), in the greenhouse. ARL was evaluated in all plants for nodes 2 through 6, and only a subset of plants in node 1. High and low nitrogen treatments are indicated (HN, blue; LN, red).

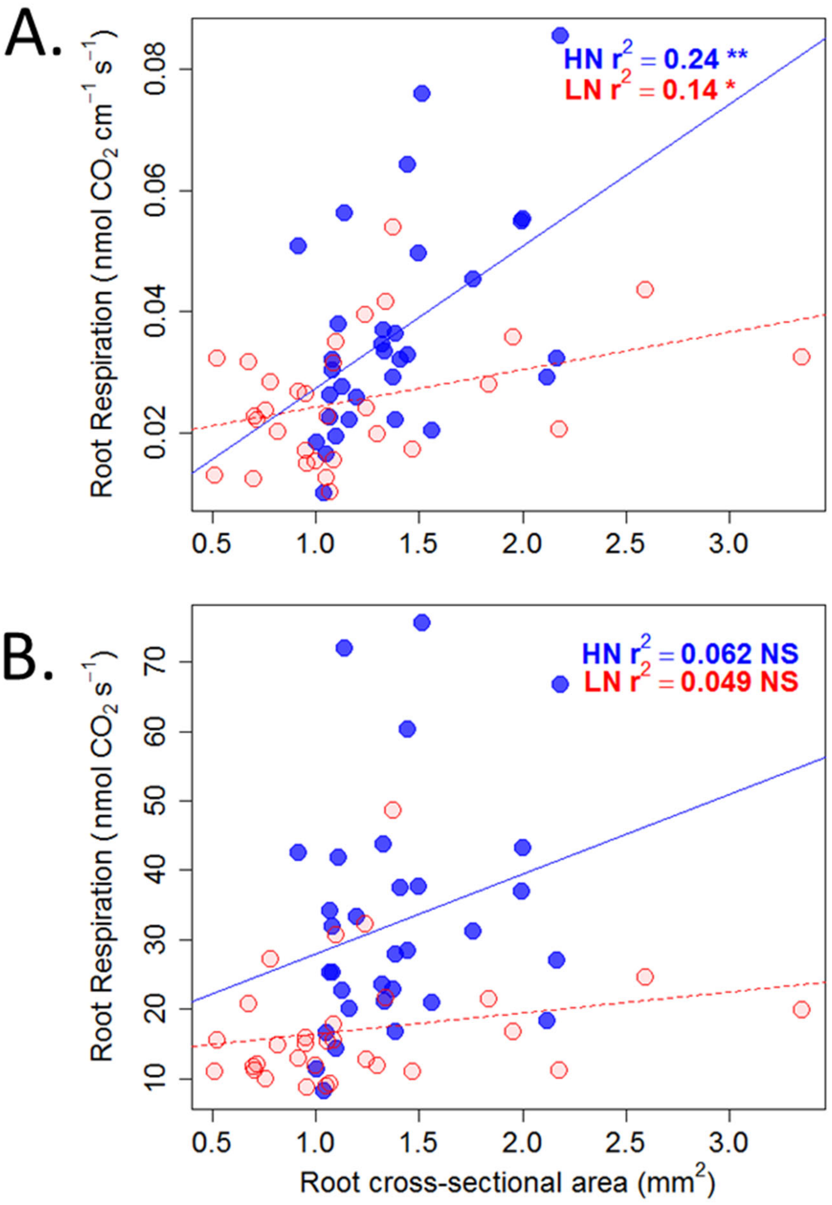

Figure S5. Relationship between axial root diameter and respiration among maize RILs. Linear regression of root cross-sectional area (RXA) averaged from second and third node roots against (A) root respiration per unit root length averaged from three roots each from nodes 2 and 3, and (B) total axial root respiration (root respiration rate multiplied by axial lengths of all developed nodes except the first node), from individual plants of maize IBM RILs grown in high (HN, blue) or moderate low nitrogen (LN, red) treatments in the greenhouse. R^2 value and significance ($p< 0.05$*, 0.01**, $p>0.1$ NS, not significant) are indicated.
