# Supplementary material for: Nodal root diameter and node number in maize (Zea mays L.) interact to influence plant growth under nitrogen stress
Source: Plant Direct. 2021 Mar 16;5(3):e00310. doi: 10.1002/pld3.310 (PMC7963125; doi:10.1002/pld3.310)
Supplement: Supplementary file 2 — Table S1‐S3 [file PLD3-5-e00310-s002.docx]

**Table S1.** **Plant materials**

| **Germinated** | **26-Sep-15** | **14-Jun-15** |
| --- | --- | --- |
| **Root Sampling** | **November 3-4, 2015** | **3-Sep-15** |
| **Soil Coring** | **NA** | **9-Sep-15** |
|  | **GH2** | **PA15** |
|  | IBM30 | IBM30 |
|  | IBM126 | IBM59 |
|  | IBM178 | IBM126 |
|  | IBM201 | IBM129 |
|  | IBM277 | IBM178 |
|  | IBM323 | IBM181 |
|  | IBM352 | IBM201 |
|  | IBM181 | IBM277 |
|  |  | IBM323 |
|  |  | IBM352 |
|  |  | IBM365 |

**Table S2.** **Nutrient Solution used for fertigation in greenhouse mesocosm experiments.**

|  | **High nitrogen (HN)** | **Low Nitrogen (LN)** |
| --- | --- | --- |
| **Element** | **μM** | **μM** |
| NO3 | 6500 | 130 |
| NH4 | 80 | 10 |
| P | 500 | 500 |
| Mg | 2000 | 2000 |
| S | 3500 | 4500 |
| Ca | 3500 | 2310 |
| K | 3010 | 1500 |
| Cl | 10 | 500 |
| B | 14 | 14 |
| Mn | 3 | 3 |
| Zn | 1 | 1 |
| M | 0.5 | 0.5 |
| Cu | 0.4 | 0.4 |
| Fe | 110 | 110 |

**Table S3.** **Nitrogen stress levels across experiments.** Percent reduction refers to average across genotypes. Range refers to range among genotypes.

| Study | No. of genotypes | Metric | % Reduction | Range |
| --- | --- | --- | --- | --- |
| Greenhouse | 8 | Dry shoot mass | 46 | 29-60% |
| Field | 11 | Dry shoot mass | 30 | 15-55% |
|  |  | Yield | 20 | 12-31% |
